# Lower Posterior Cingulate N-Acetylaspartate to Creatine Level in Early Detection of Biologically Defined Alzheimer’s Disease

**DOI:** 10.3390/brainsci12060722

**Published:** 2022-05-31

**Authors:** Qianyun Chen, Jill Abrigo, Wanting Liu, Elyia Yixun Han, David Ka Wai Yeung, Lin Shi, Lisa Wing Chi Au, Min Deng, Sirong Chen, Eric Yim Lung Leung, Chi Lai Ho, Vincent Chung Tong Mok, Winnie Chiu Wing Chu

**Affiliations:** 1 Department of Imaging and Interventional Radiology, Faculty of Medicine, The Chinese University of Hong Kong, Prince of Wales Hospital, Hong Kong SAR, China; maxinecqy@link.cuhk.edu.hk (Q.C.); jillabrigo@cuhk.edu.hk (J.A.); dkyeung@cuhk.edu.hk (D.K.W.Y.); shilin@cuhk.edu.hk (L.S.); b119551@cuhk.edu.hk (M.D.); 2 Department of Medicine and Therapeutics, Faculty of Medicine, The Chinese University of Hong Kong, Prince of Wales Hospital, Hong Kong SAR, China; lauyuenting1110@163.com (W.L.); elyiahan@cuhk.edu.hk (E.Y.H.); lisaau@cuhk.edu.hk (L.W.C.A.); vctmok@cuhk.edu.hk (V.C.T.M.); 3 Department of Nuclear Medicine & Positron Emission Tomography, Hong Kong Sanatorium & Hospital, Hong Kong SAR, China; sirong.chen@hksh.com (S.C.); eric.yl.leung@hksh.com (E.Y.L.L.); garrettho@hksh.com (C.L.H.)

**Keywords:** Alzheimer’s disease, magnetic resonance spectroscopy, N-acetylaspartate

## Abstract

Alzheimer’s disease (AD) was recently defined as a biological construct to reflect neuropathologic status, and both abnormal amyloid and tau are required for a diagnosis of AD. We aimed to determine the proton MR spectroscopic (^1^H-MRS) patterns of the posterior cingulate in biologically defined AD. A total of 68 participants were included in this study, comprising 37 controls, 16 early AD, and 15 late AD, who were classified according to their amyloid and tau status and presence of hippocampal atrophy. Compared with controls, early AD showed lower N-acetylaspartate (NAA)/creatine (Cr) (*p* = 0.003), whereas late AD showed lower NAA/Cr and higher myoInositol (mI)/Cr (all with *p* < 0.05). Lower NAA/Cr correlated with a greater global amyloid load (*r* = −0.47, *p* < 0.001) and tau load (*r* = −0.51, *p* < 0.001) and allowed a discrimination of early AD from controls (*p* < 0.001). Subgroup analysis showed that NAA/Cr also allowed a differentiation of early AD from controls in the cognitively unimpaired subjects, with an area under the receiver operating characteristics curve, sensitivity, and specificity of 0.96, 100%, and 83.8%, respectively. Lower posterior cingulate NAA levels may help to inform underlying neuropathologic changes in the early stage of AD.

## 1. Introduction

Alzheimer’s disease (AD) is the most common type of dementia and results in synapse loss and neuronal death. Early detection could allow a potential intervention and facilitate recruitment in clinical trials [1], but establishing a diagnosis is hindered by histopathologic evidence of amyloid plaques and tau tangles in the brain in post mortem examinations [2,3]. This has driven the research impetus for the identification of in vivo imaging biomarkers to aid in AD diagnosis. Structural brain imaging may show hippocampal volume loss, but this occurs in the late stage of the disease and is not specific, as it can also be age-related [4] and present in non-AD forms of dementia [5]. Positron emission tomography (PET) is currently the best surrogate investigation, as it allows for the in vivo visualization of β-amyloid (Aβ) and pathologic tau. However, drawbacks, such as the limited availability of radioactive tracers and expensive cost, hinder its widespread utilization [6].

Proton magnetic resonance spectroscopy (^1^H-MRS) is a magnetic resonance imaging (MRI) technique that allows non-invasive and radiation-free in vivo interrogation of the neurochemical composition of biological tissues. It provides regional measurements of brain metabolites that serve as markers of neuronal density, cellular turnover, and energy metabolism, making it a sensitive tool for the detection of early disease. ^1^H-MRS has been reported in clinically diagnosed dementia [7], and metabolite changes have been reported to precede clinical symptomatology and inform prognosis [7,8].

Approximately 10% to 30% of clinically diagnosed AD dementia cases do not display AD pathologic changes at autopsy [9]. On the other hand, 30% to 40% of cognitively unimpaired (CU) elderly persons have AD pathologic changes at autopsy [10]. To better reflect the neuropathologic underpinnings of AD, a biomarker classification system has been recommended to define AD in living persons [11], incorporating biomarkers of Aβ deposition (A), pathologic tau (T), and neurodegeneration (N). According to the A/T/N system, individuals with biomarker evidence of Aβ alone are considered to have Alzheimer’s pathologic change, while biomarker evidence of both Aβ and pathologic tau are required for a diagnosis of AD. Most previous ^1^H-MRS studies focused on clinically diagnosed AD; however, the MRS pattern in biologically defined AD is poorly understood. In this study, we aimed to investigate the ^1^H-MRS patterns in the spectrum of biologically defined AD based on the ATN scheme. Furthermore, we explored the association between MRS metabolites and Aβ and tau burden, as well as the diagnostic performance of ^1^H-MRS in the detection of abnormal AD pathologies in the early stage.

## 2. Materials and Methods

### 2.1. Subjects

This is a retrospective analysis of data from a prospective study. Participants were recruited under the Chinese University of Hong Kong—Screening for Early AlzhEimer’s Disease Study (CU-SEEDS), which seeks to explore imaging biomarkers to facilitate early AD diagnosis. Informed consent was obtained from all subjects involved in the study. We included males and females, aged 50–80 years, of Chinese ethnicity, and with no diagnosis of neurological disorders who underwent PET and MRI within a 3-month interval. Their age, sex, presence of cognitive impairment (using Hong Kong List Learning Test (HKLLT) [12]), and Montreal Cognitive Assessment (MoCA) scores (Hong Kong version) [13] were recorded. Each subject was assigned one of two cognitive labels as cognitively unimpaired (CU) or cognitively impaired (CI) based on their HKLLT Z-scores. Subjects with a score greater than −1 were CU; otherwise, they were CI.

### 2.2. Biomarker Group Classifications

To explore the MRS pattern in the spectrum of biologically defined AD, we grouped subjects based on the ATN scheme recommended by National Institute on Aging and Alzheimer’s Association (NIA-AA) in 2018 [11]. Aβ alone determines if individuals have Alzheimer’s pathologic change (A+T−), and both Aβ and pathologic tau are required for a diagnosis of AD (A+T+). Neurodegeneration is considered to occur in the later stage. Therefore, the enrolled cohort was categorized into the following groups: controls (A−T−N−), AD-at-risk (A+T−N−), early AD (A+T+N−), and late AD (A+T+N+). 

A/T/N status was determined by PET and MRI examinations, and binary results from these examinations were obtained from radiologists’ reports with the following criteria: (1) A+, abnormal increased ^11^C-PIB uptake as visually observed and reported as positive by the radiologist and/or global retention > 1.41; (2) T+, abnormal increased ^18^F-T187 uptake as visually observed and reported as positive by the radiologist and/or global SUVR > 1.13; and (3) N+, the presence of mesial temporal lobe atrophy (MTA) as visually scored on coronal T1-weighted MRI images [14]. Atrophy was considered present for an average score of ≥1.5 in subjects younger than 75 years and of ≥2 in subjects aged 75 years and older [15]. All PET and structural MRI scans were independently interpreted by a nuclear medicine specialist (E.Y.L.L., 20 years’ experience) and a neuro-radiologist (J.A., 10 years’ experience), who were blinded to the subjects’ cognitive and respective imaging data.

### 2.3. PET Acquisition and Analysis

An amyloid PET scan was conducted using ^11^C-Pittsburgh compound B (PIB) tracer, and brain imaging was acquired at 5 and 35 min after intravenous injection. A tau PET scan was conducted using an ^18^F-flortaucipir [T807] tracer, and images were acquired at 0 min and 85 min after T807 injection.

The contours of regions-of-interest (ROI) were automatically created from computed tomography (CT) images and propagated to PET images by the quantitative software tool MIMneruo (version 4.1, MIM Software Inc., Cleveland, OH, USA). Then, the global tracer retention was calculated by taking the mean standardized uptake value ratio (SUVR) from the ROIs, including the gyrus rectus, lateral and medial temporal lobe, frontal gyrus, occipital lobe, posterior cingulate gyrus, precuneus, putamen, thalamus, caudate, and superior parietal lobule, with brainstem as reference.

### 2.4. MRI Acquisition and Analysis

MRI was performed using a 3T scanner (Philips Achieva TX, Best, The Netherlands) with an eight-channel head coil. The structural MRI image was acquired using a 3D T1-weighted volumetric sequence with the following parameters: echo time (TE) = 3.5 ms, repetition time (TR) = 7.5 ms, flip angle = 8°, field of view (FOV) = 250 × 250 × 171 mm^3^, acquired resolution = 1.1 × 1.1 × 1.2 mm^3^, and SENSE acceleration factor = 2. Structural MRI images were segmented using FreeSurfer v6.0 (http://surfer.nmr.mgh.harvard.edu/ accessed on 1 August 2021) to obtain hippocampal volume. The volumes were normalized and reported as fractions relative to total intracranial volume prior to statistical analysis. 

### 2.5. ^1^H-MRS Acquisition and Analysis

**Figure 1:**
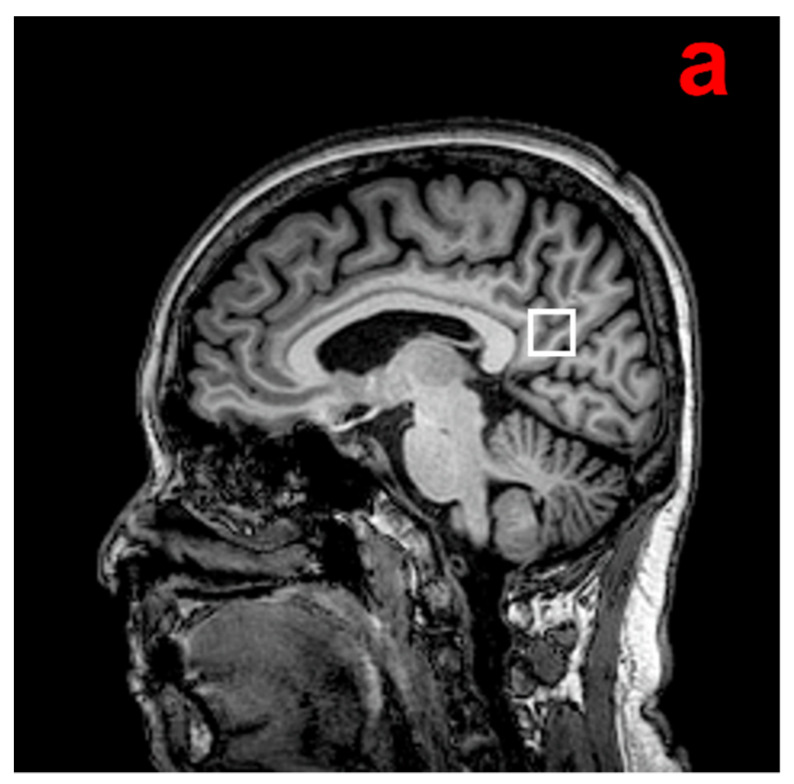

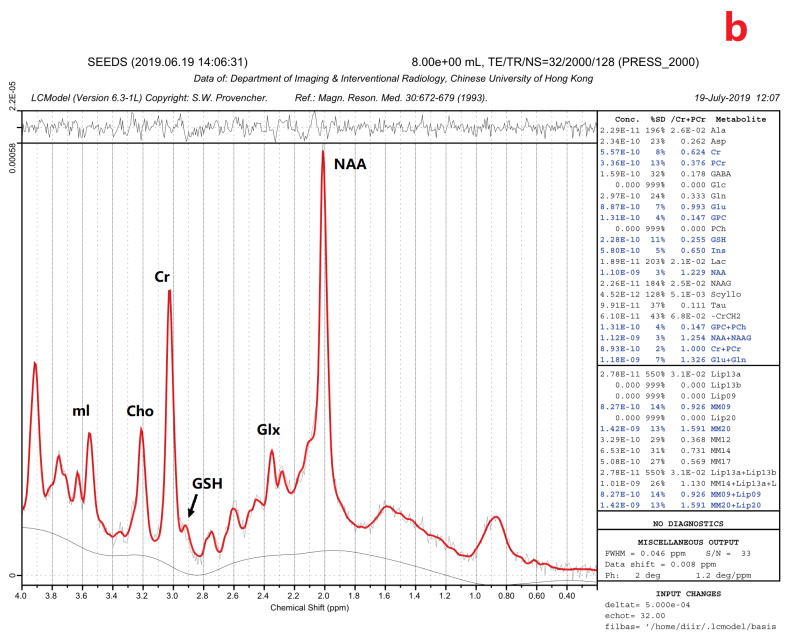
(**a**) Example of the ^1^H-MRS voxel localization. (**b**) MR proton spectra. mI, myoInositol; Cho, choline-containing compound; Cr, creatine; GSH, glutathione; Glx, glutamate and glutamine; NAA, N-acetylaspartate; ppm, parts per million [17].

^1^H-MRS examination was performed using the point-resolved spectroscopy (PRESS) sequence (TR = 2000 ms, TE = 32 ms, bandwidth = 2000 Hz, 1024 data points, 128 excitations, acquisition time = 4 min 52 s). An 8 cm^3^ (2 × 2 × 2 cm) voxel that mainly included the posterior cingulate cortex (PCC)/precuneus region was manually placed on a midsagittal T1-weighted image, including the right and left PCC (Figure 1). This region demonstrates early metabolic reduction over the course of AD [16]. It is also a well-defined midline structure from which spectra can be acquired with high reproducibility and high quality [7].

The quality of each MR spectrum was visually assessed by an experienced physicist (D.K.W.Y.) who was blinded to the clinical condition. Peak areas of metabolites were calculated by a fully user-independent program, Linear Combination of Modeled spectra (LCModel) [17]. Metabolites with a fitting error of <15% calculated by the LCModel were included; therefore, the following metabolites were collected for the analysis: *N*-acetylaspartate (NAA), myoInositol (mI), glutathione (GSH), glutamate and glutamine (Glx), choline-containing compound (Cho), and total creatine (creatine + phosphocreatine, Cr). Metabolite levels were expressed as a ratio relative to Cr, which has a relatively stable peak area [18].

### 2.6. Statistical Analysis

The normal distribution of data was confirmed with the Shapiro–Wilk test. Group differences were tested using one-way analysis of variance (ANOVA) followed by Scheffe’s tests for post hoc comparisons of normally distributed continuous variables; otherwise, a Kruskal–Wallis test along with a Mann–Whitney U test was conducted for variables with skewed or unknown distribution. Chi-square tests were employed to analyze categorical variables.

To understand the underlying causes of the observed abnormalities in the metabolite ratios, we studied their associations with the global occurrence of amyloid and tau. Association between metabolite ratios, Aβ, and tau load were determined using partial correlation (denoted as *r*), controlling for age and sex.

To evaluate the diagnostic performance, receiver operating characteristic (ROC) analysis was performed to discriminate between the following groups: controls versus early AD; controls versus late AD; and early AD versus late AD. Hippocampal fraction (HF) was included for comparison as it is the most established biomarker in AD. To explore the performance of metabolite ratios in detecting early AD in individuals without overt cognitive symptoms, we also performed a ROC analysis in the CU group. Diagnostic performance was evaluated using the area under the ROC curve (AUC). The optimal cutoff for positivity was determined using the Youden index [19]. Then, accuracy, sensitivity, and specificity were calculated based on the derived cutoff value. DeLong’s test [20] was performed to compare differences in the performance of various diagnostic metrics. All statistical analyses were performed using SPSS 26.0 (Armonk, NY, USA). Statistical tests were two-sided, with a significance level set to *p* < 0.05.

## 3. Results

### 3.1. Demographics

A total of 71 subjects were included in this study, comprising 37 control subjects, 3 subjects with AD-at-risk, 16 subjects with early AD, and 15 subjects with late AD. The AD-at-risk group was excluded from further analysis due to small sample size. Demographic details are presented in Table 1. The different groups were comparable in terms of sex and years of education (*p* = 0.46 and *p* = 0.78, respectively) but differed in age, HKLLT Z-score, MoCA score, global PIB retention, T807 SUVR, and hippocampal fraction (all with *p* ≤ 0.001). 

### 3.2. ^1^H-MRS Results

**Figure 2:**
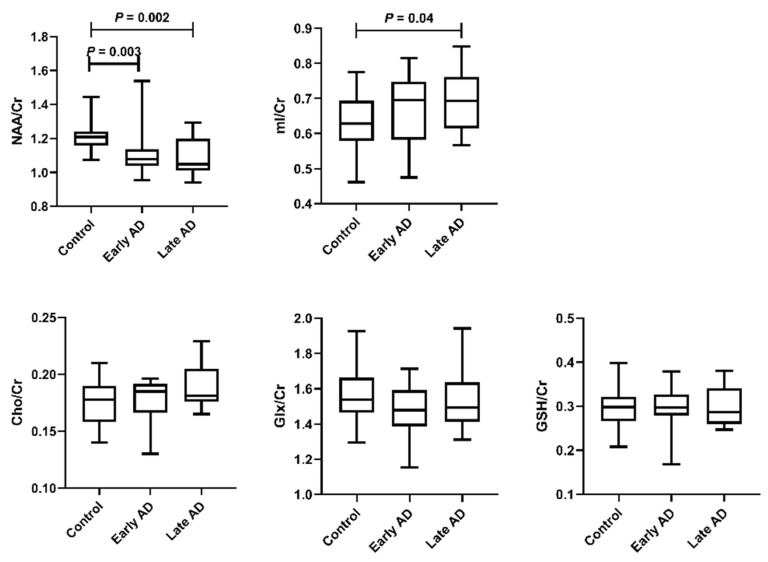
Comparison of MRS metabolite levels across groups. The bars show the median and inter-quartile range. NAA/Cr was significantly different between the control and early AD groups and between the control and late AD groups. mI/Cr was significantly different between the control and late AD groups.

Figure 2 shows comparisons of the MRS metabolite ratios among the three groups. Compared with controls, early AD showed lower NAA/Cr (*p* = 0.003), and late AD showed lower NAA/Cr (*p* = 0.002) and higher mI/Cr (*p* = 0.04). Cho/Cr, Glx/Cr, and GSH/Cr were comparable across groups (*p* = 0.10, *p* = 0.22, and *p* = 0.86, respectively).

### 3.3. Correlations of ^1^H-MRS Metabolite Ratios with Aβ and Tau

Metabolite ratios that showed significant group differences were further analyzed to investigate their correlation with Aβ and tau. The results are shown in Table 2. A lower NAA/Cr level correlated with greater PIB retention (*r* = −0.47, *p* < 0.001) and T807 SUVR (*r* = −0.51, *p* < 0.001), while a higher mI/Cr correlated with greater PIB retention (*r* = 0.39, *p* = 0.001) and T807 SUVR (*r* = 0.47, *p* < 0.001).

### 3.4. ROC Analysis

**Figure 3:**
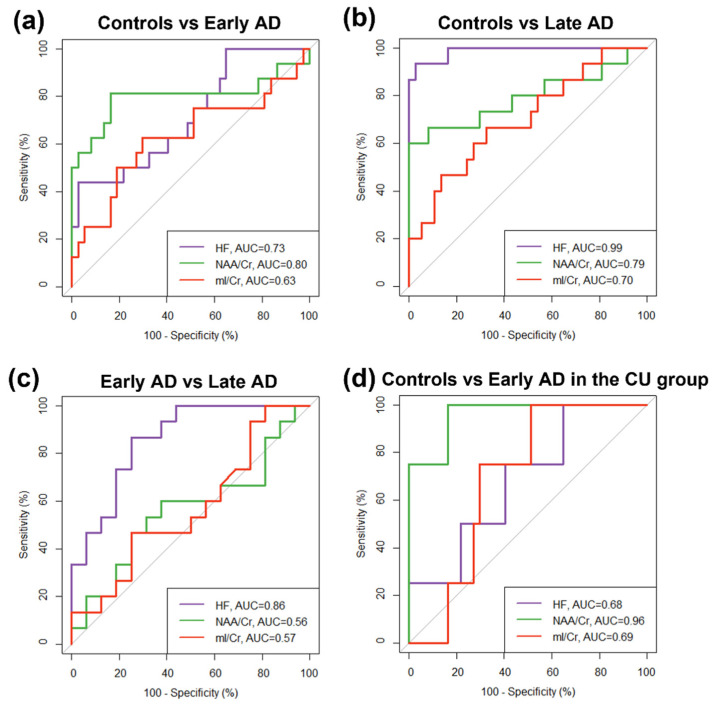
ROC curves of different metrics (hippocampal fraction (HF), NAA/Cr and mI/Cr) for the classification of (**a**) control vs. early AD groups, (**b**) control vs. late AD groups, (**c**) early AD vs. late AD groups, and (**d**) control vs. early AD in 41 cognitively unimpaired (CU) subjects. The area under the ROC curve (AUC) for each metric is shown. All metrics allowed a differentiation between the control and late AD groups. Only HF allowed a differentiation between the early AD and late AD groups, whereas only NAA/Cr allowed differentiation between the control and early AD groups. NAA/Cr also allowed the detection of early AD in the CU group.

Figure 3 and Table 3 show the results of the ROC analysis of NAA/Cr, mI/Cr, and HF to discriminate between the control, early AD, and late AD groups. The discrimination between control and late AD groups was achieved by all metrics, with AUC up to 0.99 (*p* < 0.001) for HF, followed by 0.79 (*p* < 0.01) for NAA/Cr, and 0.70 (*p* < 0.01) for mI/Cr, where HF was significantly better than NAA/Cr (*p* = 0.02) and mI/Cr (*p* < 0.001). The discrimination between early AD and late AD groups was only achieved with HF (AUC = 0.86, *p* < 0.01, sensitivity = 86.7%, specificity = 75.0%), while the discrimination between control and early AD groups was only achieved with NAA/Cr (AUC = 0.80, *p* < 0.001, sensitivity = 81.3%, specificity = 83.3%). In addition, NAA/Cr also allowed the discrimination between control and early AD (AUC = 0.96, *p* < 0.01, sensitivity = 100%, specificity = 83.8%) in the CU group.

## 4. Discussion

The present study investigates the MR spectroscopic pattern in biologically defined AD and its potential role in early AD detection. In the early AD (A+T+N−) group, a significant reduction in NAA/Cr was observed in the PCC, which enabled the distinction of early AD from normal controls (A−T−N−). Moreover, NAA/Cr significantly decreased with the severity of either amyloid or tau burden. Notably, NAA/Cr allowed the discrimination of early AD from controls in the CU group. All these observations imply that lower NAA in the PCC may indicate neuropathologic changes in the early stage of AD.

The lower NAA/Cr and higher mI/Cr of the PCC in biologically defined AD are in agreement with previously documented NAA and mI change patterns in clinically diagnosed AD [7,21,22]. NAA is considered a marker of neuronal number, density, and variability, and the decrease in its level indicates a loss of neuronal integrity [23]. It is also considered to be closely related to mitochondrial energy metabolism [24]. As such, a lower NAA/Cr may reflect neuronal loss and energy decline in the PCC. The significant reduction in NAA level is supported by Wong et al. [21]. They performed ^1^H-MRS in a higher magnetic field (7T) with a tissue concentration technique to remove bias from cerebrospinal fluid (CSF), thus giving a sensitive and accurate estimation of the NAA concentration. The reduced NAA detected by a 3T scanner in our study shows the accessibility for clinical use. In addition, our participants were grouped by ATN biomarkers, allowing us to explore the ^1^H-MRS pattern in the ATN spectrum of AD. The reduction in NAA/Cr in the early AD group reveals that NAA/Cr levels change before medial temporal atrophy.

NAA/Cr showed a good discriminative ability in classifying control and early AD groups, indicating that changes in NAA levels may parallel abnormal Aβ and tau and precede hippocampal atrophy. However, the wide range of AUC values may not encourage its clinical use. NAA/Cr also achieved excellent performance in detecting early AD in the CU group, suggesting the potential of NAA/Cr in detecting preclinical AD. Given the small and imbalanced sample size for the classification in the CU group (4 early AD vs. 37 controls), these results are preliminary, and further validation is needed for larger cohorts. In addition, considering that classification in the CU group is between subjects with A−T−N− and subjects with A+T+N−, it remains unclear whether it is the correlation between NAA/Cr and Aβ or the correlation between NAA/Cr and tau that contributes to the classification performance. 

As for mI/Cr, mI is considered a marker of glial activity or neuroinflammation, and its increase in AD has been attributed to the activation of mI-rich astrocytes and microglia [25]. Significantly elevated mI/Cr was observed only in the late AD groups, indicating a later step in AD pathogenesis, although whether it precedes hippocampal atrophy remains unclear. However, mI abnormality is suggested as an early event in the progression of AD pathology, as elevated mI/Cr was observed in APOE ε4 carriers compared with noncarriers in CU subjects with normal CSF Aβ42 levels [26]. We observed elevated mI/Cr in the early AD group, but the elevation was not significant. Our sample size may be too small to show an effect. Given the insignificant elevation in mI/Cr in the early AD group, changes in NAA/mI were not investigated, although NAA/mI is commonly reported in the literature as it showed the strongest association with pathologic measures [27].

For Cho/Cr, we did not observe significant differences across biologically defined AD groups. The Cho peak in ^1^H-MRS represents glycerophosphocholine and phosphocholine. These are the breakdown products of phosphatidylcholine from myelin and cell membranes, and they also serve as precursors of acetylcholine, which is important for synaptic transmission. Higher Cho/Cr in AD is proposed to be a consequence of cell membrane catabolism to compensate for the cholinergic deficit in disease progression. An elevation in Cho in AD was observed in other studies [28,29]. Our sample size may have been too small to show this effect.

For Glx/Cr, in line with Kantarci et al. [30], we did not observe significant changes, although their decrease has been reported in AD [31,32,33]. Currently, the interpretation of Glx/Cr is challenging due to limitations imposed by the technique, as glutamate and glutamine levels are commonly measured together as a composite peak owing to their poor spectral resolution. Glx forms a continuous cycle mediating glutamatergic neurotransmission. Although more than 40% of neuronal synapses in the brain are glutamatergic, ^1^H-MRS does not appear sensitive enough to detect changes in this system.

For GSH, no significant change was found in patients with AD. GSH is considered to be an antioxidant, and thus a marker of oxidative stress. On the one hand, its increase can be a compensatory response to reactive oxygen species generated by amyloid and tau [34], while its decrease could suggest a depletion in the decompensated state [35]. Theoretically, an increase followed by a decrease would be expected in the AD spectrum, as observed in our cohort; however, the changes did not reach statistical significance. A larger cohort and longitudinal investigation might yield further mechanistic insights into the brain’s response to oxidative stress in AD. 

This study has several limitations. First, our sample size is small, limiting observations across the ATN spectrum of AD. There were only a few A+T−N− subjects, precluding their inclusion in the analysis. Such is the challenge when using our stringent classification criteria in adhering to the biological definition of AD. Second, ^1^H-MRS was performed on a single voxel placed in the region of PCC, where contamination from CSF can influence spectral results. We adopted this method as it is a widely accepted practice in dementia imaging [30], and it is in line with our aim to test the technique for clinical application. Third, the classification results between control and early AD in the CU group are preliminary due to the small and imbalanced sample size of the two groups. Further validation is needed in a large cohort. Finally, we limited the analysis of metabolites as ratios relative to Cr, finding their levels stable across cohorts, and thus serving as an ideal internal reference metabolite.

## 5. Conclusions

Our study determined the ^1^H-MRS patterns in biologically defined AD. NAA/Cr of the PCC may help inform underlying pathologic process in AD. Our results encourage further exploration of ^1^H-MRS to facilitate the early detection of AD. 

## Figures and Tables

**Table 1:** Demographic characteristics and imaging data of controls, early AD and late AD groups.

	Control (*n* = 37)	Early AD (*n* = 16)	Late AD (*n* = 15)	*p* Value
CU/CI (*n*/*n*)	37/0	4/12	0/15	<0.001 ^a,b^
Female, *n* (%)	21 (56.8%)	11 (68.8%)	11 (73.3%)	0.46
Age (years)	64.9 (6.4)	66.9 (8.9)	70.5 (5.5)	0.04 ^b^
Education (years)	9.9 (3.7)	10.7 (1.1)	9.4 (1.1)	0.78
HKLLT *Z*-score	0.25 (−1.08, 0.71)	−1.80 (−2.19, −0.91)	−2.03 (−2.24, −2.03)	<0.001 ^a,b^
MoCA	25.58 (3.55)	21.81 (5.88)	15.07 (4.61)	<0.001 ^b,c^
Global PIB retention	1.26 (0.05)	1.58 (0.17)	1.68 (0.20)	<0.001 ^a,b^
Global T807 SUVR	1.03 (0.07)	1.23 (0.22)	1.27 (0.19)	<0.001 ^a,b^
Hippocampal fraction (%)	0.58 (0.09)	0.50 (0.07)	0.40 (0.05)	<0.001 ^b,c^

Values are expressed as mean with standard deviation for normal distribution data, otherwise expressed as median with interquartile range. AD: Alzheimer’s disease; CU: cognitively unimpaired; CI: cognitively impaired; HKLLT: Hong Kong List Learning Test; MoCA: Montreal Cognitive Assessment score; PIB: ^11^C-Pittsburgh compound B tracer; T807: ^18^F-flortaucipir; SUVR: standardized uptake value ratio. ^a^ Statistically significant difference between controls versus early AD (*p* < 0.05); ^b^ Statistically significant difference between controls versus late AD (*p* < 0.05); ^c^ Statistically significant difference between early AD versus late AD (*p* < 0.05).

**Table 2:** Association between MRS metabolite ratios and PIB retention and T807 SUVR in the whole sample of participants.

	Global PIB Retention		Global T807 SUVR	
	*r*	*p*	*r*	*p*
NAA/Cr	−0.47 *	<0.001	−0.51 *	<0.001
mI/Cr	0.39 *	0.001	0.47 *	<0.001

* Correlation is considered significant at the 0.05 level (2-tailed). PIB: ^11^C-Pittsburgh compound B tracer; T807: ^18^F-flortaucipir tracer; SUVR: standardized uptake value ratio.

**Table 3:** Performance of metabolite ratios in classification among groups.

	AUC (95% CI)	SE	*p* Value	Optimal Cutoff †	Accuracy (%)	Sensitivity (%)	Specificity (%)
Control vs. Early AD (*n*/*n* = 37/16)							
HF	0.72 (0.56–0.87)	0.08	0.01 *	0.49	81.1	43.8	97.3
NAA/Cr	0.80 (0.63–0.97)	0.09	<0.001 *	1.14	83.0	81.3	83.8
mI/Cr	0.63 (0.45–0.81)	0.09	0.14	0.67	67.9	62.5	70.3
Control vs. Late AD (*n*/*n* = 37/15)							
HF	0.99 (0.96–1)	0.01	<0.001 *	0.45	96.2	93.3	97.3
NAA/Cr	0.79 (0.62–0.96)	0.08	<0.01 *	1.07	88.5	60.0	100
mI/Cr	0.70 (0.54–0.86)	0.08	0.02 *	0.66	67.3	66.7	67.6
Early AD vs. Late AD (*n*/*n* = 16/15)							
HF	0.86 (0.73–0.99)	0.07	<0.01 *	0.44	80.7	86.7	75.0
NAA/Cr	0.56 (0.35–0.77)	0.11	0.11	1.07	61.3	60.0	62.5
mI/Cr	0.57 (0.36–0.77)	0.11	0.51	0.73	61.3	46.7	75.0
Control vs. Early AD in the CU group (*n*/*n* = 37/4)							
HF	0.68 (0.43–0.94)	0.13	0.24	0.60	41.5	100	35.1
NAA/Cr	0.96 (0.88–1)	0.04	<0.01 *	1.14	85.4	100	83.8
mI/Cr	0.69 (0.51–0.87)	0.09	0.27	0.63	53.7	100	48.6

† Youden index-derived cutoff; * Significant at *p* < 0.05 level; AUC, area under the receiver operating characteristic curve; SE, standard error; CI, confidence interval; HF, hippocampal fraction; CU, cognitively unimpaired.

## Data Availability

The data supporting the findings of this study are available from the corresponding author on reasonable request.
